# Resilience and Resource Management

**DOI:** 10.1007/s00267-015-0582-1

**Published:** 2015-07-14

**Authors:** Eleanor D. Brown, Byron K. Williams

**Affiliations:** Science and Decisions Center, U.S. Geological Survey, 12201 Sunrise Valley Drive, Reston, VA 20192 USA; The Wildlife Society, 5410 Grosvenor Lane, Suite 200, Bethesda, MD 20814 USA

**Keywords:** Resilience, Resource management, Threshold, Uncertainty

## Abstract

Resilience is an umbrella concept with many different shades of meaning. The use of the term has grown over the past several decades to the point that by now, many disciplines have their own definitions and metrics. In this paper, we aim to provide a context and focus for linkages of resilience to natural resources management. We consider differences and similarities in resilience as presented in several disciplines relevant to resource management. We present a conceptual framework that includes environmental drivers, management interventions, and system responses cast in terms of system resilience, as well as a process for decision making that allows learning about system resilience through experience and incorporation of that learning into management. We discuss the current state of operational management for resilience, and suggest ways to improve it. Finally, we describe the challenges in managing for resilience and offer some recommendations about the scientific information needs and scientific issues relevant to making resilience a more meaningful component of natural resources management.

## Introduction

Among and even within disciplines, there are numerous definitions of resilience that focus on different attributes or different perspectives (e.g., Klein et al. 2004; Folke 2006; Zhou et al. 2010). The use of the term has grown over the past several decades, so that by now, many areas of research and application have their own definitions, metrics, and discipline-specific literature. From its beginnings in engineering and materials science in the nineteenth century, where resilience was seen as a measure (the “modulus of resilience”) of the elastic deformation of materials under physical strain, the resilience concept has expanded into other disciplines. It was applied in ecology in the 1970s, then spread into the social sciences, especially in connection with social impacts of disasters and natural hazards, and now is referenced broadly with respect to any change or adverse circumstance. Yet after more than 40 years of academic research and debate, there is not common agreement on a definition of resilience, or on how to measure it, test it, and manage for it.

Our objectives in this paper are to provide a context and focus for linkages of resilience to natural resources management. We consider differences and similarities in the resilience concept as defined in several disciplines and clarify the basis for managing any system of interest. We use a conceptual framework that includes environmental drivers, management interventions, and system responses cast in terms of system resilience, as well as a process for decision making. Regardless of the definition of resilience, better decision making is promoted by a decision process that provides managers with information about what actions will contribute to resilience, what attributes to measure, how to learn about system resilience through experience, and how to incorporate that learning into management. We discuss the current state of resilience management and suggest some ways to improve it. Finally, we highlight challenges in managing for resilience, including some of the scientific information needs and the scientific issues relevant to operational management of resources to enhance resilience.

## Approaches to Resilience

Definitions and treatments of resilience tend to separate along disciplinary lines, based on the nature of the system and its drivers. Some variants of the resilience concept that are most relevant to natural resources include: (1) *ecological resilience*, with a focus on the response of ecological systems to shocks; (2) *social*–*ecological system resilience*, focusing on the response of social–ecological systems to shocks, on the basis of the perspective that social and ecological systems are linked; and (3) *disaster resilience*, focusing on responses of social structures and relationships to disasters or natural hazards. There is overlap in the concepts, focus issues, and methodologies of these various perspectives (Table 1), but some useful generalizations can be made. In this section, we compare these variant concepts and examples of relevant research on specification and measurement of the system of interest, which are basic to building a decision framework for management of system resilience.

**Table 1:** Comparison of the resilience concept among several disciplines

	Conceptual elements typically emphasized	Typical approach	Typical methods	Important research focus
Ecological resilience	Alternative stable states Thresholds Regime shift Critical transition Hysteresis Slow & fast variables Adaptive capacity	Natural sciences	Empirical observation Natural science methods including mathematical modeling & experimentation	Identification of alternative stable states Identification of key drivers and system response variables Early warning of approach to thresholds between alternative system states
Social–ecological system resilience	Ecological elements (as above) Adaptive cycle Transformability Social capital Social networks Learning Governance Vulnerability Panarchy	Social sciences Natural sciences	Empirical observation Natural science methods Social surveys, statistical analysis & other social science methods No social experimentation	Linkage of complex social and ecological systems, often emphasizing either social entity or ecological entity Identification of key drivers and system response variables
Disaster resilience with social focus or social–ecological focus	Hazard Vulnerability Risk Preparedness Mitigation Socioeconomic attributes Social networks Institutions Infrastructure	Social sciences Natural sciences (for hazards) Engineering sciences (for infrastructure)	Empirical observation Natural science methods Social surveys, statistical analysis & other social science methods No social experimentation Civil engineering	Speed with which given social systems or sectors can return to normal function after a disturbance Magnitude of disturbance that infrastructure can resist

### Ecological Resilience

**Fig. 1:**
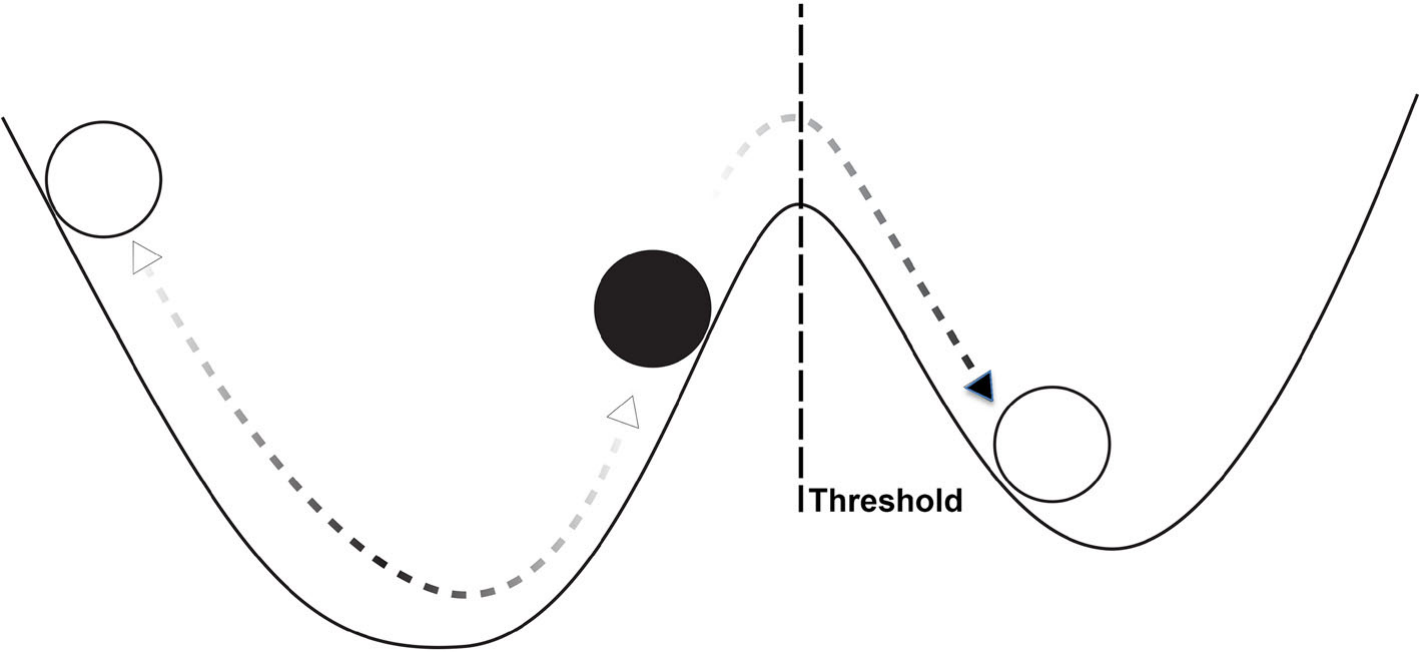
Illustration of the resilience concept, in which the state of a system fluctuates over a range of possible states in response to system processes and external perturbations. In ecological resilience, the system may have more than one alternative stable state (i.e., dynamical regime). The system state (represented as a ball) tends to remain in one of the alternative regimes (represented as a basin) unless a perturbation is severe enough to move the system past a threshold into an alternative state. There may be multiple alternative stable states and multiple stable and unstable equilibria. After Liao (2012)

As originally developed in an ecological context by Holling (1973), the ideas of system dynamics were applied to population dynamics and other behaviors of ecological systems, in contrast to the then prevailing view that stable equilibria in ecological systems were the norm. Some of the numerous interpretations of the resilience perspective in ecology are described by Folke (2006). A specific definition of ecological resilience is* the magnitude of disturbance that a system can tolerate before it shifts into a different state (stability basin) with different controls on structure and function* (Scheffer 2009). Some relevant definitions (Scheffer 2009) include the following: alternative stable states are two or more contrasting dynamical regimes in which the system can maintain itself, under the same external conditions. A threshold is a boundary between two alternative regimes. In a critical transition, a system is pushed over a threshold resulting in a self-propagating shift to an alternative regime. Hysteresis is the tendency for a system’s persistence in one of the alternative regimes. Slow variables and fast variables are the different classes of drivers that govern system change. Finally, adaptive capacity is the degree to which a system is capable of reorganization and adaptation (Table 1). Figure 1 shows a system with multiple stable states, such that the system can move into different states, with different equilibria, over time as conditions change. The focus in ecological resilience is on “persistence, change, and unpredictability” (Holling 1996).

Work on ecological resilience rests on a body of well-developed theory, and quantitative theoretical models often provide the basis for empirical testing and experimentation. Research on the resilience of ecosystems often focuses on transitions between system states across thresholds and on features of the alternative states (dynamical regimes) where the system tends to remain unless perturbed. Mathematical models, especially system-dynamics approaches like simulations that allow for transitions between alternative regimes, are widely used to predict system behavior and to address large-scale temporal or spatial processes such as climatic cycles and evolutionary trends. In some systems, repeated transitions can be observed directly, as in lakes and rangelands (Scheffer et al. 2009), and experimentation is possible with closed systems or small parts of larger systems.

Empirical studies have documented multiple alternative regimes and abrupt transitions across thresholds in numerous real-world natural systems. In most of these systems, the slow and fast variables that drive system change have been identified and measured, sometimes after decades of research. Examples span numerous species and ecological and natural systems at various scales. These include semi-arid rangelands, where shifts between grass and woody shrub regimes are due to pastoral grazing pressure (Walker et al. 1981; Rietkerk and Van de Koppel 1997); pest cycles in boreal forests, where periodic spruce budworm outbreaks kill maturing evergreen forests as foliage becomes denser and hides budworms from avian predators (Holling 1973); shallow lakes, where regime shifts between clear and turbid states are driven by nutrient inputs (reviewed by Scheffer 2009); North Atlantic cod stocks, where abundant populations collapsed in the 1990s after overfishing and never recovered (Bundy et al. 2010); and paleo-climatic cycles driven by variables such as atmospheric carbon dioxide levels and changes in the Earth’s orbit, which have resulted in alternation between warm and cool conditions multiple times during Earth’s history (reviewed by Scheffer 2009).

Recent empirical work has taken a number of different directions. Some recent studies have focused on identifying the variables that signal change in a particular system. For example, Lindegren et al. (2012) analyzed multiple methods and monitoring datasets in “hindcasting” a recent Baltic Sea transition in order to test their utility in signaling the transition. Other studies focused on temporal data, such as fluctuations shown in time series of physical or biological variables. For example, Schooler et al. (2011) analyzed natural time series data to create a model of alternative stable states of invasive *Salvinia* plant population dynamics and predict the state during which control of the plants by weevils would be most effective. In an approach involving spatial data, Hirota et al. (2011) investigated forest response to climate stressors by analyzing remotely sensed imagery to demonstrate evidence for multiple stable tree distribution states and sharp transitions related to precipitation amounts.

The search for generic, universal indicators that a system is approaching a threshold is an active topic of both theoretical and empirical research. Most work has been in ecology and climate science (reviewed by Scheffer et al. 2009, 2012), where knowledge of the conditions that could “tip” the climatic system into an alternative regime might help to avoid irreversible changes. Much of the work on thresholds involves generic changes in a system’s structure and dynamics as it nears a threshold, for example, changes in the degree of heterogeneity and connectivity of components (e.g., as shown in size distributions of vegetation patches (Kefi et al. 2007)), and changes seen in time series (e.g., “critical slowing down” of the system’s recovery from disturbance (Van Nes and Scheffer 2007)). In two real-world examples, Dakos et al. (2008) analyzed time series of eight ancient events of climate change reconstructed from geological records to test for early signs of upcoming abrupt shifts to an alternate climatic state, while Carpenter et al. (2011) experimentally caused a whole-lake ecosystem transition by manipulating the food web in order to analyze biological and physical variables for early signs anticipating the transition.

In sum, research on ecological resilience tends to center on analysis of a natural or ecological system’s alternative stable states, the drivers of transitions across thresholds between states, and the characteristics of system structure and dynamics when approaching a threshold. Improved management would follow from models that accurately predict thresholds and management actions to avoid thresholds.

### Social–Ecological System Resilience

As the ecological resilience perspective began to spread outside ecology into the social sciences, subsequent efforts to integrate the social and ecological dimensions took many directions (see Folke’s (2006) review). The Resilience Alliance, a research network, promotes the view that social and ecological systems are interlinked as complex systems that influence each other. A definition of resilience that includes both is as follows: *resilience is the capacity of a system to absorb disturbance and reorganize while undergoing change, so as to retain essentially the same function, structure, identity, and feedbacks* (Walker and Salt 2006). According to Carpenter et al. (2001), the dimensions of resilience in social–ecological systems are (1) the amount of disturbance a system can absorb and still remain within the same stable state; (2) the degree to which the system is capable of self-organization; and (3) the degree to which the system can build and increase the capacity for learning and adaptation. When applied to social systems, some of the language borrowed from the ecological concepts takes on different meanings. For example, the adaptive capacity of an ecological system depends on diversity of species, and in some cases on rapid evolution, whereas in a social system, it depends on cultural diversity, learning, and innovation.

The concept of social–ecological system resilience is frequently framed in terms of a core heuristic model (“panarchy” (Gunderson and Holling 2002)) of system change. An adaptive cycle of change involves four distinct phases, which are complicated by interactions of system behaviors among different temporal scales or different spatial scales, or both. Transformability refers to the capacity to create a fundamentally new system. The panarchy model posits that resilience ebbs and flows at different parts of the adaptive cycle, an idea that is relevant to the timing and scale of effective interventions (e.g., Holling 2001).

Research on resilience of social–ecological systems often involves the capacity for system functions to persist and adapt in response to a disturbance. In general, studies tend to emphasize either ecological systems with some social factors included, or more commonly, social systems with some ecological aspects added in. Social factors tend to be related to anthropogenic drivers of system transitions (e.g., economic aspects of fertilizer use that drives lake eutrophication cycles (Carpenter et al. 2001)). Although social resilience studies typically use language borrowed from system-dynamics and complexity theory, in most cases, resilience is used simply as a metaphor (Carpenter et al. 2001). Mathematical modeling is so far mostly absent in research on structural change or transitions in societal systems (Timmermans et al. 2008). Discussion of transitions tends to remain qualitative, as compared to research on ecological transitions. Methods include those of the social sciences, with natural resource data often included as important elements.

Developing conceptual models of social–ecological systems, and metrics to make resilience measurable, are important topics in this literature. In an example of the study of ecological systems with social factors included, Carpenter et al. (2001) built on decades of ecological work to propose models of adaptive cycles of lake/agriculture and rangeland/pastoral systems that included socioeconomic indicators such as the price of the fertilizer that drives lake eutrophication. Investigations based mainly on the study of social systems with ecological factors included are numerous, and span diverse communities, resource sectors, institutions, or geographic regions. For example, at the community level, Marshall and Marshall (2007) presented metrics based on a conceptual model of social resilience of commercial fishing communities in coastal Australia, while Cinner et al. (2009) studied livelihood diversity, capacity to organize, and governance in communities near Madagascar’s marine protected areas. Baral and Stern (2011) estimated stocks of natural and social capital in Nepali communities in the Annapurna Conservation Area. At the institutional level, Gupta et al. (2010) proposed metrics for studying the role of institutions in enabling the adaptive capacity of Dutch society in response to climate change. At the regional level, Walker et al. (2009) conducted a qualitative watershed-level, synthetic “regional resilience assessment” in the Goulbourn–Broken catchment, Australia, identifying likely biophysical, economic, or social thresholds, plus main issues, drivers, and potential shocks and system responses. In another qualitative, synthetic regional assessment, Cumming et al. (2005) studied the southwest Amazon and developed a conceptual process to identify key networks, pinpoint the variables that serve as system drivers, and assess the potential for the system to cross a threshold.

In sum, studies of resilience in social–ecological systems often seek to define and synthesize a system framework (usually with the main emphasis on either ecological or social aspects), describe important variables, and derive associated quantitative or qualitative metrics of system states. Description of alternative states, identification of key social variables, and forecasting of system responses would help to improve decision making.

### Disaster Resilience

“Disaster resilience” is related to the responses of a social entity to disasters or natural hazards. Here, we consider two thrusts of disaster resilience work, one that focuses almost solely on social aspects and one that has incorporated the ideas about social–ecological systems.

#### Social Aspects

In the branch of resilience work focusing more or less exclusively on social structures and relationships, a typical definition of resilience is as follows: *the ability to prepare and plan for, absorb, recover from, and more successfully adapt to adverse events* (National Research Council 2012). The enhancement of resilience is held to allow for better anticipation of disasters and better planning to reduce disaster losses (National Research Council 2012). The social focus can include communities, economies, institutions, individuals, employment or industrial sectors, and so on.

**Fig. 2:**
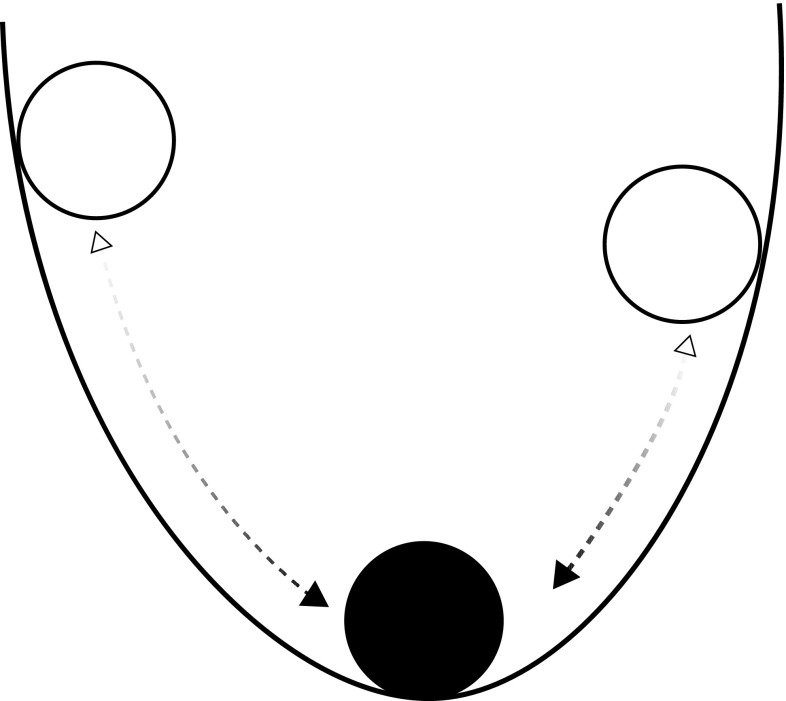
Engineering resilience, in which the system state (ball) tends toward a single, stable equilibrium within a single stable state (i.e., dynamical regime). After Liao (2012)

In general, this research is concerned with (1) how quickly or efficiently particular social subsectors or subsystems can return to a “normal” condition after a disturbance, or (2) the magnitude of disturbance that infrastructure can resist and remain unchanged. Liao (2012) pointed out that this view of social resilience is akin to the “engineering resilience” defined by Holling (1996), which emphasizes “efficiency, constancy, and predictability” (Holling 1996). In the context of engineering resilience (Fig. 2), resilience usually relates to a single stable state of a system, and how quickly the system returns to that state after disturbance (e.g., Liao 2012). The methodology used is typically that of the social sciences, or of engineering or materials science for built infrastructure. Natural science information such as earthquake magnitude, wind force, or storm surge potential is used insofar as it is relevant to prediction, prevention, preparedness, response, and recovery.

Identifying social or infrastructural vulnerability due to various hazards and assessing risks are major topics of research in the disaster resilience literature. How to quantify resilience is a key question in this context, because without numerical methods, it is “impossible to monitor changes or to show that community resilience has improved” (National Research Council 2012). Many efforts at quantification tend to include the aggregation of numerous variables into some sort of multi-metric index. For example, the SoVI social vulnerability index (from work by Cutter et al. (2003)), a commercially available statistically derived metric for comparing capacity for preparation and response at the county and sub-county level, synthesizes 32 variables based on census data and expert judgments in a principal components analysis. In a study developing another index for social resilience, Cutter et al. (2010) constructed a composite resilience indicator for communities (BRIC) that includes social, economic, institutional, infrastructure, and community resilience sub-indices. In other approaches to measuring community- and local-level social resilience, Harrald (2012) proposed a logic framework with measures of inputs, outputs, activities, and outcomes, for developing quantifiable community resilience metrics based on socioeconomic indicators, social networks, and emergency response capabilities. Berke et al. (2012) developed a set of principles to guide improvement of federally required state hazard mitigation plans and rate the quality of their components (such as hazard, vulnerability, capability and risk assessments; mitigation policies; monitoring; and coordination with local governments). Some investigations are focused on particular hazards or vulnerabilities. For example, the SPUR method (San Francisco Planning & Urban Research Association 2008) measured specific performance objectives for earthquake recovery in the Bay area, while the Argonne National Laboratory Resilience Index (Fisher et al. 2010) was developed for vulnerabilities in critical infrastructure and produced an index value from expert evaluation of different categories of critical infrastructure facilities along multiple dimensions (security management, information sharing, protective measures).

In sum, research in the context of disaster and hazard resilience tends to focus on conceptualizing and measuring the speed and efficiency with which a social entity could potentially regain its original (or desired) stable state after a disturbance; or the magnitude of disturbance that it can resist. Clearly framing the objectives, defining the management alternatives, and evaluating consequences of various alternatives could help determine the most effective management decisions.

#### Social–Ecological Aspects

The social–ecological system concept of resilience is now used in much interdisciplinary work on disaster and hazards research, according to Klein et al. (2004), who reviewed its emergence in the disaster literature. The Hyogo Framework for disaster risk reduction (UN International Strategy for Disaster Reduction 2007) considers resilience as the ability of a community or society exposed to hazards to resist, absorb, accommodate, and recover in a timely and efficient manner. Their definition of resilience is *the capacity of a system, community, or society to resist or to change in order that it may obtain an acceptable level in functioning and structure.* This is determined by the degree to which the social system is capable of organizing itself and the ability to increase its capacity for learning and adaptation, including the capacity to recover from a disaster (UN International Strategy for Disaster Reduction 2004).

In this branch of the literature, the primary emphasis is also on social attributes, with added ecological aspects. The focus is on how, and how quickly, specific subsectors can reconstitute function, or reorganize social or physical infrastructure, in the face of disturbance. This view of disaster resilience emphasizes Holling’s (1996) “ecological” aspects (persistence, change, unpredictability) rather than “engineering” aspects of constancy and predictability (Liao 2012). Studies usually include environmental or natural resource aspects. Climate-related hazards such as droughts, flooding, or storms are important topics, forming a natural connection to the topic of climate change and climate change adaptation. In this context, the frequently used term “climate resilience” refers to the resilience of a social entity to climate change, as opposed to the resilience of the climate system itself to disturbances.

System models often focus on specific hazards or vulnerabilities and include interdisciplinary work on human/natural interactions or social and cultural responses to the surrounding environment. For example, Klein et al. (2004) reviewed coastal mega-cities and weather-related hazards, including effects of human activities in modifying hazard potential, socioeconomic risks in different sectors (water, agriculture, health), and climate change adaptation. Adger et al. (2005) also examined social interactions with the environment in providing an overview of social–ecological resilience to coastal disasters and discussing how governance systems can manage the interactions of social and ecological factors to cope with coastal hazards and disasters (e.g., hurricanes, tsunamis). In another approach to adapting to urban flood hazards, Liao (2012) applied resilience concepts of system persistence through changes to develop a theory about how to increase urban resilience to floods by means of new designs for flood-resistant infrastructure as an alternative to traditional flood-control construction. In a cultural study of agricultural practices in colonial Mexico, Endfield (2012) used archival data to demonstrate social learning and innovation in natural resource management, as manifested in water management practices and agricultural systems that adapted to climatic crises such as floods and droughts. In another approach to the agriculture sector, Zhou et al. (2010) used a case study of farm resilience to drought in northern China to propose a geographically based conceptual model for analyzing and measuring community disaster resilience.

In sum, research in this context of disaster and hazard resilience often highlights the linkage of social and ecological systems. A common methodological element is interdisciplinary work on human/natural interactions or social responses to the environment including natural resources management, as a means of increasing social adaptive capacity. Learning to use natural resource management to mitigate hazards more effectively would lead to better management decisions.

## Federal Agencies and Resilience

The near ubiquity of the resilience concept and the proliferation of research in different disciplines suggest that management for system resilience will eventually make its way into the mainstream and become widely adopted. This should lead to advances in resource management, because better knowledge of how systems work is a necessary basis for improving management. As pointed out by Benson and Garmestani (2011), the understanding that ecological systems are characterized by multiple stable states, with transitions among them that can be abrupt and governed by non-linear dynamics, has obvious implications for natural resource management. Environmental management that proactively avoids thresholds, so that the system does not shift (perhaps irreversibly) into another state with different structures and processes, will be more effective in sustaining the resources being managed. One indication of where operational management for resilience stands at present is the state of resilience efforts in federal agencies in the United States. These agencies collectively have broad mandates to manage millions of acres of publicly owned land, resources, social institutions, and disaster response capabilities.

Resilience has emerged as a fashionable term among federal policymakers, managers, and scientists. Website searches of federal agencies involved in resource or disaster management (e.g., the U.S. National Oceanographic and Atmospheric Agency/National Marine Fisheries Service [NOAA/NMFS], U.S. Forest Service, Federal Emergency Management Agency [FEMA], U.S. Department of the Interior [DOI]) return thousands of results, and the number is growing rapidly. NOAA (e.g., NOAA 2010) and the Forest Service (e.g., USDA Forest Service 2013) have been explicit about policy incorporating both ecological and social resilience into their management activities (Benson and Garmestani 2011), whereas FEMA (e.g., FEMA 2014) uses the term more vaguely. For example, NOAA’s next-generation strategic plan (NOAA 2010) places resilience in the forefront, as shown by the following statement: “NOAA’s vision of the future: resilient ecosystems, communities and economies… Resilient ecosystems can absorb impacts without significant change in condition or function… A vision of resilience will guide NOAA and its partners in our collective effort to reduce the vulnerability of communities and ecological systems in the short term, while helping society avoid or adapt to potential long-term environmental, social, and economic changes.” The Forest Service Manual (USDA Forest Service 2014) contains an interim policy directive (FSM 2020) entitled “ecological restoration and resilience,” which states that the aim of the directive “is to reestablish and retain ecological resilience of National Forest System lands and associated resources…” (see also Office of the Federal Registrar 2014). In the FEMA 2011–2014 strategic plan, priority number one is to “strengthen the nation’s resilience to disasters.” Stated policies notwithstanding, however, there is currently limited operational management by federal agencies for resilience of ecological or social systems, especially at larger scales. Reasons for this include insufficient understanding of complex social–ecological systems, a lack of quantified metrics for measuring resilience, incompatibility of resilience thinking with existing institutional frameworks and management goals, inadequate legal and regulatory frameworks, and a lack of funding (Benson and Garmestani 2011; Allen et al. 2011).

On the other hand, agencies do continue to make scientific progress by building on what is currently known about system dynamics and resilience. Agencies are pursuing substantive research about conceptual aspects of social and ecological resilience, and investigating ways to apply these aspects in management. For example, the Forest Service (USDA Forest Service 2013) and NOAA (Jepson and Colburn 2013) are both studying social resilience of natural resource–dependent communities. Ecosystem resilience concepts are being incorporated into information on new forest practices in the Sierra Nevada (North 2012). Both NOAA and the U.S. Geological Survey (USGS) have active research in relevant areas like paleo-climate, paleo-ecology, ecosystem processes, and climate processes. NOAA partnered with the National Science Foundation on Comparative Analysis of Marine Ecosystem Organization (CAMEO), a program with scientific research themes grounded in system-dynamics concepts related to the resilience of social–ecological systems (CAMEO Science Steering Committee 2012). (Unfortunately CAMEO fell victim to Congressional “budget sequestration” funding cuts and was ended in 2012.) With respect to disaster resilience, USGS and NOAA have historically provided natural hazards science to aid management of social preparedness, response, and recovery.

## Improving Resilience Management

Although efforts in the management of resilience thus far have been limited, general trends in the federal agencies in resilience thinking are encouraging and offer an opportunity for advances in understanding resilience and its management. But a great deal of work (and commitment of resources) will be needed over an extended period of time for real progress into be made in operationalizing the management of resilience. In this section we offer recommendations for improving the situation, including a synthetic framework for making management decisions.

### Resilience Framework

**Fig. 3:**
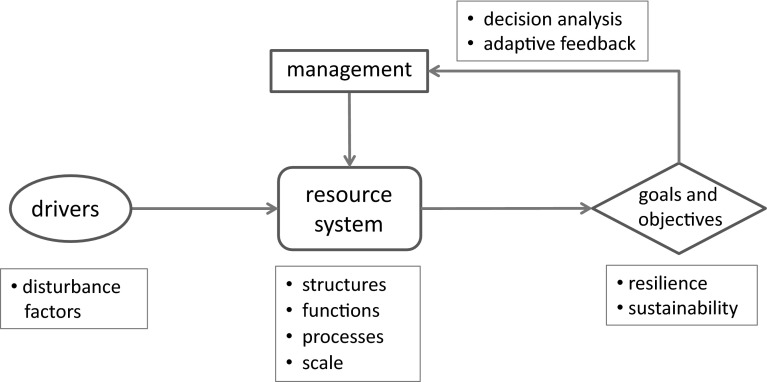
Framework for the management of resilience. Resource systems are influenced by management and other external drivers, as well as internal resource processes. In combination, these factors affect sustainability and resilience, which in turn can inform future management actions

A framework for resilience can be described in a systems analytic context that includes decision making with management objectives (Williams and Brown 2012) specified in terms of system resilience (Fig. 3). Ultimately decision making, and in particular decision making that targets resilience, concerns “what to do, where, and when” (Wilson et al. 2007). We believe these questions can best be approached through a careful structuring of resilience as a decision problem (Arvai et al. 2001; Possingham 2001; Wilson et al. 2006; Gregory et al. 2012). Such an approach can be thought of as “a formalization of common sense for decision problems which are too complex for informal use of common sense” (Keeney 1982).

Several elements are required for decision making to be based on more than intuition or chance. For example, the process should include some criterion for measuring the relative value of decision alternatives, and a mechanism for selecting among them. This is true whether or not the primary concern of decision making is the maintenance of system resilience. The relevant systems, whether social, ecological, or physical, are thought of as at least potentially dynamic, with changes that occur as a result of internal processes (such as reproduction, mortality, and movement of organisms in ecosystems), external events (such as weather conditions, earthquakes, and floods in hazards assessment), and/or human interventions (such as landscape fragmentation, resource removals, and pollutant inputs in social–ecological systems). Thus, the decision framework includes a specification of the system, the identification of alternative choices for its management, the recognition of consequences, and a mechanism for deciding which alternative to choose under different circumstances. Often the process is framed in terms of uncertainties about system status and process, and embedded in a social milieu of multiple stakeholders and decision makers with competing objectives.

Of course, problem framing is only a part of the overall process. Understanding how potential decision outcomes are perceived and valued by stakeholders is a key to the development of objectives, which in turn influence all other aspects of a decision assessment (Keeney 1992; Arvai et al. 2001). For resilience, systems can be characterized by uncertainties as to system responses to environmental drivers and interventions. This means that the models used to project the consequences of actions and their valuation must accommodate both the potential for change and associated uncertainties. Yet another important feature of the process is the tracking of change through monitoring, which can play several critical roles (Williams et al. 2002). For example, monitoring is required for estimates of system status that fold into state-dependent decision making and is needed to assess the degree to which management objectives are being met. It provides a basis for learning, through the comparison of predictions against observations. Monitoring also provides the data needed to update the models used in decision making. In the particular context of resilience, it is important to monitor not only the system of interest, but also the variables that drive system change. A special challenge is the effect of cross-scale influences, whereby the dynamics of a system at a given scale of interest are influenced by variables operating at both larger and smaller scales. Cross-scale factors can account for changes in resilience and contribute to a propensity highlighted in panarchy theory for systems to undergo periodic transformation and reconfiguration (Gunderson and Holling 2002). Depending on the time frame and rate of change of the cross-scale factors, in order to account for the dynamics of systems with that propensity, it may be necessary to track cross-scale factors and model their influence on the system. This can add considerably to the complexity of a decision analysis.

### Integrating Resilience and Decision Making

In some instances, there has been a tendency to view a decision-oriented approach as antithetical to resilience theory, or at least as an alternative perspective in natural resources management (Walker et al. 2002; Fischer et al. 2009; McFadden et al. 2011; Polasky et al. 2011). In particular, resilience proponents have been critical of decision making that seeks to maximize resource exploitation (Walker and Salt 2006; Norberg et al. 2008). Such an approach is held to reduce spatial, temporal, or organizational heterogeneity that would otherwise limit exploitation, and thereby lead to the homogenization of a system and make it less able to cope with unexpected change and disturbance (Meyer 1976; Holling and Meffe 1996). On the other hand, decision analysts have been critical of resilience theory for not providing much practical advice about how actually to manage for resilience. A source of tension between the two perspectives is the pursuit of efficiency in maximizing the productivity of one or a few resource components, rather than attempting to keep options open by maintaining and enhancing diversity (Johnson et al. 2013). Although this tension has been pronounced in assessments of the management of ecological and social–ecological systems, it can perhaps be resolved by making resilience, rather than efficient exploitation, the specific objective of the decision process (Fig. 3).

A decision-oriented approach to managing for resilience has various implications. A learning-based approach such as adaptive management, which accumulates knowledge about system responses to management, is a necessity because managing for resilience is so complex (Gunderson 2010). With resilience as an objective, management must focus on the system drivers and management interventions that can influence long-term system viability (Zellmer and Gunderson 2009). A “resilience orientation” suggests that decision making should be more attuned to the potential for systems to change, and to uncertainties that underlie the risks of unintended outcomes. For example, the recognition of thresholds of abrupt change separating alternative system states makes it important to account for them in decision making, in order to reduce the likelihood that some disturbance will shift the system into a less desirable regime from which it would be difficult to recover. Quantifying resilience is crucial, because measurable and testable metrics are needed to define objectives, monitor change, and evaluate management actions as part of the decision-making process. Further, if resilience is to become a regulatory mandate, quantified metrics are needed to provide “clearly articulated and enforceable standards” (Benson and Garmestani 2011) that agencies could use in evaluating management alternatives. Willingness to explore social dynamics and manipulate social components (for example, water allocations in Western rivers) would ultimately be an important aspect of managing for resilience of coupled social–ecological systems (Benson and Garmestani 2011).

## Future Research Directions

An important but largely unmet need for resilience management is scientific information. In a natural resource context, this might mean the science needed for managing social and ecological systems, as well as the natural hazards information needed for managing social resilience in the face of disasters. For example, human interventions in conserving, harvesting, and regulating biological resources are linked to diversity, productivity, and resilience of ecological and social systems, and knowledge of these linkages is a key requisite for managing ecosystems and understanding how regulations affect human use patterns and ecological systems (CAMEO Science Steering Committee 2012). A useful area of research is the integration of ecological factors in assessment of social resilience to natural hazards, especially where ecosystem management can influence the scope and risk associated with hazards. Social resilience to natural hazards (“disaster resilience”) also requires scientific prediction, measurement, and monitoring of hazards for social preparedness and response.

In addition to data needs, a number of scientific issues must be addressed in developing practical approaches to the management of resilience. Synergistic recommendations have been made about how to operationalize management for resilience at various scales and levels (e.g., Carpenter et al. 2001; Chapin et al. 2009; Allen et al. 2011; Walker and Salt 2012; Johnson et al. 2013). For example, a key challenge in problem framing is to determine the appropriate spatial and temporal boundaries of a social–ecological system. The emphasis on multiple scales in resilience thinking has tended to make the bounding of decision problems more difficult and sometimes intractable (Levin 2000;Walker et al. 2002; Fischer et al. 2009; Polasky et al. 2011). Empirical methods are needed for forecasting outcomes, which involves the formulation of models of resilience and an accounting of uncertainties (Carpenter et al. 2001; Scheffer and Carpenter 2003; Groffman 2006; Scheffer 2009). The traditional focus on single populations needs to be expanded to include ecosystems as well as human interactions. Models focusing on system processes with potentially abrupt regime shifts (Johnson et al. 2013) are needed, as well as new methods of linking multiple quantitative models and new ways to reduce the uncertainty related to more complex models (CAMEO Science Steering Committee 2012). Much attention is being given to monitoring in terms of methods for detecting early warning signs of abrupt regime changes (e.g., Karunanithi et al. 2008; Biggs 2009; Scheffer et al. 2009, 2012). A resilience-based approach to valuing decision outcomes has barely been explored (Carpenter et al. 1999; Peterson et al. 2003), despite the need to define utility in a way that avoids a focus on a narrow range of goods or services that, if optimized, could erode resilience. Finally, decision analytic methods, particularly adaptive management, are needed for overall integration of resilience thinking into the framework of decision making in order to capture the knowledge needed to meet resilience-based objectives over time.

## Concluding Remarks

A great deal has been written about resilience, and some interesting and useful concepts have come out of the effort, though admittedly the development and application of resilience thinking have been uneven across disciplines. However, there continues to be confusion in terminology and an inadequate treatment of resilience in the context of management. In the end, our most critical needs concern how to manage for resilience: how to identify potential actions, how to measure resilience in an operationally meaningful way, how to learn about the effects of management on natural and social systems and apply that learning to improve management over time. Although increasingly urgent, these issues are largely unaddressed in any meaningful way in the literature. Our hope is that this paper can contribute to focusing discussions on them, by providing a context for the assessment and management of resilience in natural and social systems.
